# Diet Gut Microbiota Axis in Pregnancy: A Systematic Review of Recent Evidence

**DOI:** 10.1007/s13668-023-00453-4

**Published:** 2023-02-22

**Authors:** Thubasni Kunasegaran, Vinod R. M. T. Balasubramaniam, Valliammai Jayanthi Thirunavuk Arasoo, Uma Devi Palanisamy, Amutha Ramadas

**Affiliations:** Jeffrey Cheah School of Medicine and Health Sciences, Monash University Malaysia, 47500 Bandar Sunway, Malaysia

**Keywords:** Gut microbiota, Maternal nutrition, Pregnancy, Metabolism, Metagenomics

## Abstract

***Purpose of Review*:**

Although gut microbiota have been associated with the etiology of some diseases, the influence of foods on gut microbiota, especially among pregnant women, remains unclear. Hence, a systematic review was performed to investigate the association between diet and gut microbiota and their influence on metabolic health in pregnant women.

***Recent Findings*:**

We performed the systematic review using the Preferred Reporting Items for Systematic Reviews and Meta-Analyses (PRISMA) 2020 protocol to investigate the association between diet and gut microbiota and their influence on metabolic role in pregnant women. Five databases were searched for relevant peer-reviewed articles published in English since 2011. Two-staged screening of 659 retrieved records resulted in the inclusion of 10 studies. The collated findings suggested associations between nutrient intakes and four key microbes: *Collinsella*, *Lachnospira*, *Sutterella*, *Faecalibacterium*, and the Firmicutes/Bacteroidetes ratio in pregnant women.

***Summary*:**

Dietary intakes in pregnancy were found to modify the gut microbiota and positively influence the cell metabolism in pregnant women. This review, however, emphasizes the importance of conducting well-designed prospective cohorts to investigate the role of changes in dietary intakes within the pregnancy and the influence of such changes on gut microbiota.

**Supplementary Information:**

The online version contains supplementary material available at 10.1007/s13668-023-00453-4.

## Introduction

Obesity and gestational diabetes mellitus (GDM) have been established as severe public health issues and have a high risk of adverse consequences when occurring during pregnancy. Glucose and lipid metabolism-related diseases among pregnant women have repeatedly been vital indicators of several adverse maternal and established neonatal outcomes. Maternal obesity and GDM are associated with unfavourable pregnancy outcomes such as pre-eclampsia, caesarean delivery, preterm birth, and perinatal death [1–4]. Additionally, GDM raises the risk of postpartum type 2 diabetes mellitus (T2DM) in expectant mothers and the risk of obesity and other metabolic abnormalities in their offspring [5]. At 9 years following delivery, the risk of T2DM was around 20% for women with a history of GDM. Epidemiological evidence has consistently shown that among mothers with prior history of GDM, 30–84% of them had GDM recurrence in subsequent pregnancies [6], and 20–40% developed metabolic syndrome within 2–20 years [7, 8] and obesity within 5–16 years [9–11].

Prevention initiatives mainly focused on lifestyle modifications such as dietary changes and increased physical activity. Diet modifications are extensively utilized as the primary therapeutic option. Nutritional approaches for gestational hyperglycaemia share a common goal of improving blood glucose control and health outcomes for mothers and their infants. Despite the widely recognized function of medical nutrition therapy (MNT) in managing GDM, no agreement exists on the appropriate dietary nutrient advice for maintaining pregnant women’s normal glucose levels [12••], and dietary changes to prevent hyperglycaemia in pregnancy vary according to potential risk factors.

Several studies have found a substantial correlation between gut microbes and metabolic diseases [13–16]. During gestation, the gut microbiota undergoes considerable changes, which may affect the long-term metabolism of pregnant women and their newborns. The makeup of the intestinal microbiota is correlated to maternal obesity and hyperglycaemia. Various therapies, including antibiotic treatment and non-compliance intake of probiotics or prebiotic supplements, may alter metabolic function during pregnancy since all act on the gut microbiome and impact overall health [17]. The inappropriate use of antibiotics can lead to bacteria resistance, domination of microbial composition by pathogenic bacteria, loss of bacterial makeup, and decrease or even loss of certain bacterial species [18]. Hence, changes in the gut microbes might largely explain those alterations.

Diet also has been shown to alter the gut microbiota makeup within a short period [19, 20]. Studies suggest that food influence on metabolic responses differs according to the individual microbiome profile, providing a potential moderator between diet and metabolic health during pregnancy. These findings suggest that a customized strategy for human nutrition may optimize outcomes by demonstrating that a diet is not necessarily effective for all individuals or situations. The effect of specific nutritional changes on the role and makeup of gut bacteria is of great interest in the quest for the ideal strategy for preventing and managing GDM, and maternal obesity, and other metabolic disorders during gestation. Currently, very few data are available, and those that exist have conflicting findings.

Therefore, this review synthesizes recent evidence in this area. Specifically, we conducted a systematic review to examine associations between diet and gut microbiota during healthy pregnancies and pregnancies complicated by metabolic dysfunction. Additionally, we explored the possibility that specific nutrients modifying the makeup of the intestinal microbiota could have implications for pregnant women’s metabolic health.

## Methods

We used the Preferred Reporting Items for Systematic Reviews and Meta-Analyses (PRISMA) Statement 2020 [21] and the checklist (Supplementary Table S1) [21]. The systematic review protocol has been registered with the International Prospective Register of Systematic Reviews (PROSPERO) (CRD42021276459) and can be retrieved via https://www.crd.york.ac.uk/prospero/.

### Data Sources and Search Strategy

We included experimental or observational studies that reported on the role of dietary intake in modulating the gut microbiota and their impact on measures of metabolic function or glucose control (e.g. glycosylated haemoglobin (HbA1c), fasting blood glucose (FBG), oral glucose tolerance test (OGTT), insulin, Homeostatic Model Assessment for Insulin Resistance (HOMA-IR, lipid metabolism, and inflammatory profile). Studies were included if the population included pregnant women, with no exclusions based on maternal glucose levels. To be included in the review, the gut microbiota had to have been quantified from stool samples using any sequencing approach targeting the 16S ribosomal RNA gene. Studies that reported on pharmacotherapy, herbal remedies, and surgery were excluded.

We searched peer-reviewed papers from 2011 to 2022 using the following data sources: Ovid Medline, Scopus, PubMed, Cochrane Library, and Web of Science. The search strategy was built using the following MeSH terms and keywords: (gestational diabetes mellitus) OR (gestational diabetes) OR (diabetes in pregnancy) AND ((gut microbiota) OR (microbiome) OR (microbes) OR (microbial dysbiosis) OR (microbiota) AND ((diet) OR (dietary intake) OR (nutrition) OR (fats) OR (high fat diet) OR (glycemic index) OR (carbohydrate) OR (fiber) OR (fibre) OR (vitamin) OR (vegetarian) OR (fruits) OR (vegetables) OR (protein).

We limited our search to human studies and English-language publications. Additionally, we conducted manual searches for the papers using reference lists from the articles included and from earlier reviews. The complete database search strategy is provided in Supplementary Table S2.

### Study Selection

Two authors (T.K. and A.R.) eliminated duplicated studies and checked retrieved reference titles and abstracts using Endnote Version X9. Following that, T.K. and A.R. independently assessed the full texts according to the review’s eligibility. Disagreements about whether an article was eligible were resolved through discussion. Studies were considered if the following conditions were met: (1) experimental or observational studies of dietary intake among pregnant women; (2) conducted metagenomics sequencing; (3) reported maternal metabolic outcomes such as glycosylated haemoglobin (HbA1c), fasting blood glucose level, lipid profiles, or gestational weight gain; 4) published between 1 January 2011 and 5 July 2022; and (5) published in the English language. Publications not reviewed by peers, including conference proceedings, online abstracts, and book chapters, were excluded. Studies conducted among non-pregnant people with type 1 or type 2 diabetes mellitus, probiotic/prebiotic supplements, and without defined dietary patterns /nutrient composition by class or amount were also rejected.

### Data Extraction and Synthesis

T.K. and A.R. independently collected pertinent details from gathered studies using a Microsoft Excel spreadsheet: author, country, study design, number of participants, and primary and secondary outcomes. The primary outcomes were changes in the gut microbiota, including changes in relative bacterial abundance and diversity (α and β) and macro- and micronutrient levels. The nutrition levels could also be reported as a secondary outcome. Other secondary outcomes were changes in glucose–lipid metabolic profiles and weight. The data on the diverse gut microbiota and their association to metabolic variables and nutrient intakes in pregnant women, as well as the research features, were comprehensively synthesized.

### Quality Assessment and Risk of Bias

We used the National Institutes of Health (NIH) quality assessment tool for case–control, observational cohort, and cross-sectional studies to assess the included studies’ methodological quality [22]. The study population, eligibility requirements, sample size rationale, observation period, exposure and outcome data, and additional sources of bias are all considered when evaluating the study’s quality. Every considered study was given a quality rating of good, fair, or poor. Two reviewers (T.K. and A.R.) independently assessed all analyses for methodological quality.

## Results

### Study Selection and Characteristics

**Fig. 1:**
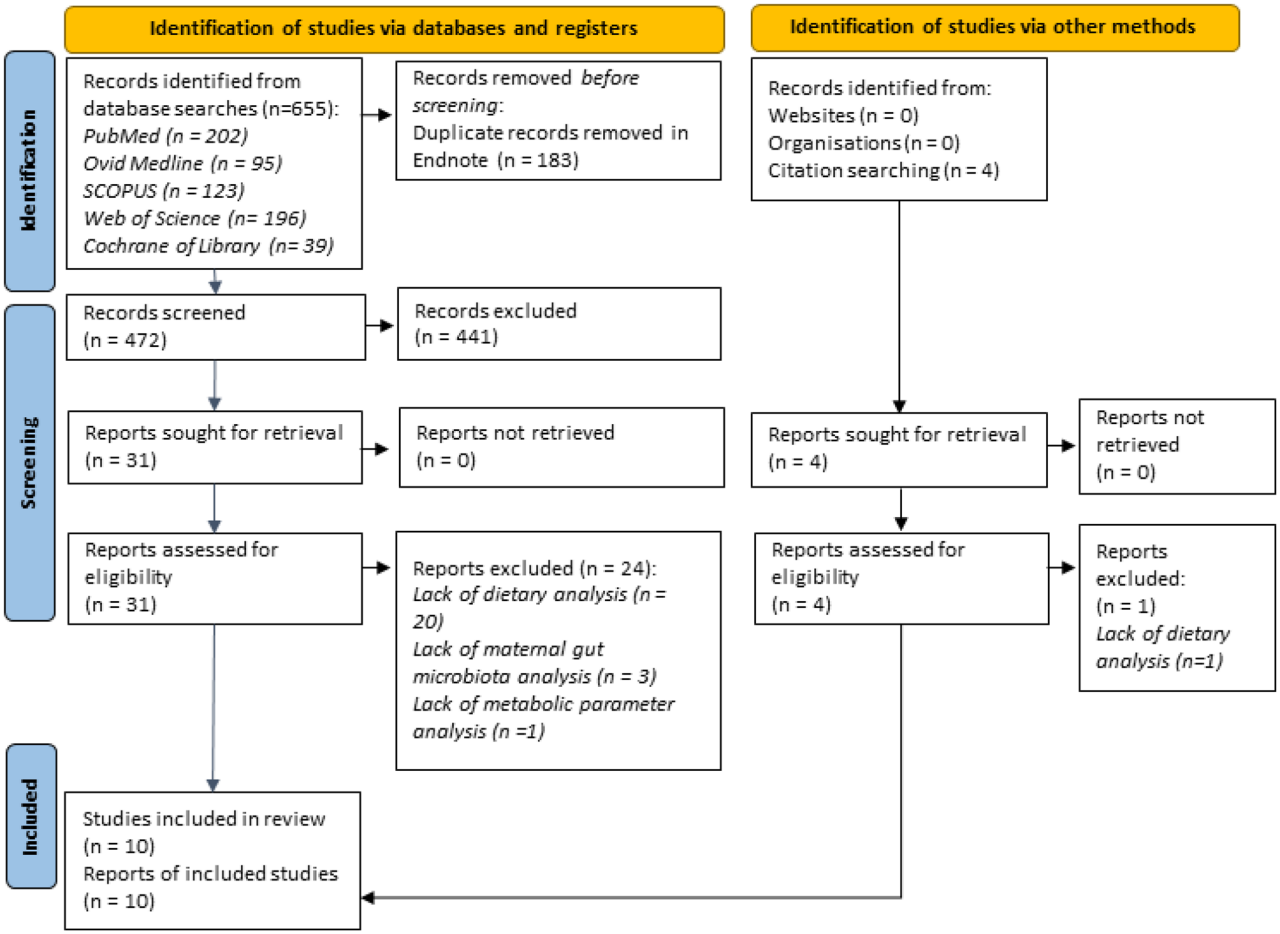
PRISMA 2020 flow chart detailing the study selection process

The database search retrieved 655 records. Four hundred seventy-two titles and abstracts were evaluated after duplicate entries of 183 articles were removed. A manual search yielded four additional records. Full texts and the reference lists of the remaining 31 records were reviewed using the eligibility criteria. Ten studies fulfilled the eligibility criteria and were included in this review (Fig. 1). The citations of excluded full texts have been listed in Supplementary Table S3.

Table 1 provides an overview of the study’s characteristics. Our search did not retrieve any experimental studies involving a dietary intervention and its influence on the gut microbiota and metabolic health of pregnant women. Two prospective cohorts [23, 24••], seven cross-sectional studies [25–29, 30••, 31], and a case–control were included in this review [32••]. Four of these studies were conducted in Australia (40%), two in China (20%), and others in Finland, Italy, Spain, and Norway. The majority of the studies had a sample size of below 100 [23, 24••, 25, 29, 30••, 31, 32••], two studies had a sample size between 100 and 150 [26, 28], and one [27] included 210 pregnant women.

**Table 1:** Summary characteristics of included studies (*n* = 10)

**Study, country **	**Study design **	**Study population **	**Mean age ± SD (years)**	**Mean BMI at booking ± SD (kg/m**^*2*^)	**Dietary intake**	**Assessment timepoints**	**Primary outcomes**	**Secondary outcomes**
Ferrocino et al. [23] Italy	PC	41 GDM	37.1 ± 4.2	ppBMI; 25.8 ± 5.9	MNT	24-28 weeks and 38 weeks	Gut microbiota	BMI, FBG, HbA1C, insulin, cholesterol, triglycerides, CRP, and nutritional analysis
Wu et al. [24••] China	PC	27 GDM, 30 non-GDM	GDM group = (32.7 ± 3.3), non-GDM = (31.4 ± 2.9)	GDM group =(71.1 ± 12.4), non-GDM = (66.9 ± 9.5)	MNT	24-28 weeks	Gut microbiota	BMI, OGTT, blood pressure
Barrett et al. [25] Australia	CS	27 pregnant women	Study arm Med= 33, control arm Med= 34	Study arm Med = 28.3, control arm Med= 28.4	Vegetarian and omnivorous diet	16 weeks	Gut microbiota	BMI, FBG, HbA1C, insulin, cholesterol, triglycerides, and nutritional analysis
Gomez-Arango et al. [26] Australia	CS	126 overweight / obese pregnant women	Overweight group = 32.0 (29.0 – 34.0), obese group = 30.5 (28.0 – 34.0)	Overweight group Med= 27.9 (27.0 – 29.1), obese group Med= 34.3 (31.8 – 41.3)	Habitual dietary intake	16 weeks	Gut microbiota; Collinsella and insulin	BMI, FBG, HbA1C, insulin, cholesterol, triglycerides, C-peptide, and nutritional analysis
Nitert et al. [27] Australia	CS	210 overweight pregnant women	High iron intake Med= 32 (29–34), low iron intake Med= 32 (27–35)	High iron intake Med= 31.6 (28·2–36·1), low iron intake Med= 31.3 (28·3–37·5)	Habitual dietary intake with pregnancy multivitamins	16 weeks	Gut microbiota and iron intake	Butanoate, lipid biosynthesis, and BMI
Roytio et al. [28] Finland	CS	100 overweight pregnant women	30.1 ± 4.7	ppBMI;30.2 ± 4.6	Habitual dietary intake	NR	Gut microbiota and dietary intake analysis	Serum lipidomic and low-grade inflammation
Mandal et al. [29] Norway	CS	60 pregnant women	NR	ppBMI22.9 ± 3.5	Habitual dietary intake	Postpartum day 4	Gut microbiota and dietary intake analysis	-
Selma et al. [30••] Spain	CS	73 pregnant women	Med=35.0 (31.0 – 36.25)	ppBMI;34.5 ± 4.3	Habitual dietary intake	Prior to giving birth in the delivery room	Gut microbiota and dietary intake analysis	Zonulin concentration
Chen et al. [32••] China	CS	74 GDM	Effective group = 30.2 ± 4.9; ineffective group = 32.2 ± 4.1	ppBMI; Effective group = 34.6 ± 4.2; ineffective group = 35.2 ± 4.9	MNT	24-28 weeks	Gut microbiota	OGTT
Robinson et al. [31] Australia	CS	22 overweight / obese pregnant women	Med=33.5 (29-38)	NR	Habitual dietary intake	16 weeks	Gut microbiota and ketonuria level	Carbohydrate intake

*PC* Prospective Cohort, *CS* Cross-sectional, *CC* Case–control, *MNT* Medical Nutrition Therapy, *Med* Median, *NR* Not Recorded, *ppBMI* Pre-pregnancy BMI, *BMI* Body Mass Index, *FBG* Fasting Blood Glucose, *HbA1C* Glycosylated Haemoglobin, *CRP* C-reactive Protein, *OGTT* Oral Glucose Tolerance Test

The age range of study participants was between 24 and 38 years old, with a BMI range of 24–35 kg/m^2^. Four studies recruited pregnant women with overweight and obesity [26–28, 31], while three recruited pregnant women diagnosed with GDM [23, 24••, 32••]. Three studies recruited pregnant women without GDM [25, 29, 30••]. Six studies included pregnant women who followed habitual dietary intake [26–29, 30••, 31], and three studies included MNT in their protocol [23, 24••, 32••]. Barret et al. compared vegetarian pregnant women with women who consumed omnivorous diets [25].

### Methodological Quality Assessment

The mean score on the NIH Quality Assessment Scale was 52.5% (50–71%). There were no studies with poor quality; seven had acceptable quality, and three had good quality (Supplementary Tables S4 and S5).

### Correlation Between Nutrients and Gut Microbial Abundance in Pregnant Women

Maternal nutrition and dietary intake are associated with maternal gut bacteria during pregnancy. Each study reported macro-/micronutrient or diet pattern associations with specific microbial makeup, relative abundances, and metabolic outcomes (Table 2).

**Table 2:** Types of gut microbiota and its association to nutrient intake in pregnant women in the included studies (*n* = 10)

**Gut microbiota**	**Normal pregnancy **[25, 29, 30••]								**Obese/overweight **[26–28, 31]				**GDM **[23, 24••, 32••]		
	Carbohydrate	Fat	MUFA	PUFA	Protein	Vitamin D	Vitamin E	Fibre	Fat	Vitamin A	Fibre	Iron	Fat	Protein	Fibre
**Proteobacteria**	↑	↓	↑	↓	↓	↑	↓								
**Bacteroidetes**	↑	↓													
**Actinobacteria**					↓										
***Faecalibacterium***														↑	
**Firmicutes**	↑	↑								↑	↑				
***Roseburia***				↑			↑								↑
***Lactobacillus***												↑			
**Ruminococcaceae**												↑			
**Lachnospiraceae**				↑					↑			↑			
***Romboutsia***	↓		↑												
***Collinsella***							↑	↓			↑				↑
***Holdemania filiformis***				↑			↑								
**Barnsiellaceae**									↓						
***Sutterella***												↑			
***Alistipes***													↑		

↑ = increased abundance; ↓ = decreased abundance. *MUFA* Monounsaturated Fatty Acid, *PUFA* Polyunsaturated Fatty Acid

#### Normal Healthy Pregnancy

All studies that included normal-weight women without GDM (normal pregnancy) reported a standard dietary intake [29, 30••], except for one study that included strict vegetarian intake [25]. Higher intake of carbohydrates in normal healthy pregnancy was positively associated with Proteobacteria [30••], *Bacteroides* [30••], and Firmicutes, but negatively linked with *Roseburia* [30••]. Barret et al. [25] noticed a richness in *Roseburia* in the vegetarian. However, there was no difference in total intake of carbohydrates or fibre between the vegetarian and omnivore groups. A lower intake of total carbohydrates was associated with a higher relative abundance of *Lachnospira* [30••].

Selma and team found that high-fat intake had higher Firmicutes [30••], *Romboutsia* (high monounsaturated fatty acids (MUFA) intake), and lower Proteobacteria [29, 30••] relative abundance in their microbiota [30••]. MUFA was also positively correlated with relative increases in Proteobacteria [29] and *Romboutsia* [30••]. *Lachnospira* also had a positive connection with total fat intake, including saturated fatty acid (SFA) and MUFA, but not polyunsaturated fatty acids (PUFA) [30••]. According to Barret et al., vegetarians consume diets high in PUFA, especially ω-6 fatty acids such as linoleic acid, compared to MUFA [25]. In their investigation, *Roseburia* richness was strongly associated with PUFA intake.

Total protein intake, especially those from an animal source, was negatively associated with Actinobacteria [30••] and Proteobacteria [29]. Proteobacteria was positively linked with vitamin D intake. However, the richness is reduced through higher vitamin E intake in healthy pregnant women [29]. Conversely, a low abundance of *Collinsella* was recorded in vegetarian women who consume a relatively high diet rich in fibre, according to Barret et al. [25] study.

#### Overweight/Obese Pregnant Women

Similar to women without metabolic risk factors, studies on overweight and obese pregnant women reported on standard diets, representative of habitual intake [26–28, 31]. Gomez-Arango et al. [26] reported that the prevalence of *Collinsella* was higher in women with low fibre consumption. Roytio et al. [28] also observed a positive correlation with Firmicutes in high-fibre/moderate-fat intake group. They also observed a low abundance of Barnsiellaceae in the high-fat intake group and a high abundance of Firmicutes strongly linked with vitamin A consumption [28].

Robinson et al. [31] found that *Roseburia* is more prevalent in the intestinal microbiota of overweight/obese pregnant women with ketonuria. However, the intake of carbohydrates did not differ significantly between women with ketonuria and those without ketonuria. The researchers speculated that it might result from the small sample population (*n* = 22).

Lastly, Roytio et al. [28] stated a significantly low abundance of *Ruminococcus* and *Roseburia* in pregnant women who consume high iron supplements. In the gut microbiota of pregnant women with minimal supplemental iron intake, bacteria generating short-chain fatty acids, including *Lachnospira*, *Sutterella*, and the lactate producer *Lactobacillus*, were dominant, according to their findings by network analyses.

#### GDM Pregnant Women

Women with GDM are generally given dietary advice based on MNT. The women with GDM recruited in the study by Wu et al. [24••] were asked to follow a diet with the target composition of 35–45% carbohydrates (80% complex carbohydrates with a low glycaemic index and 20% simple carbohydrates), 18–20% protein (50% animal and 50% plants), and 35% fat (16% monounsaturated, 10% polyunsaturated and 9% saturated) with moderately low saturated fat levels, fibre intake of at least 20–25 g/day. Acidaminococcaceae, Enterobacteriaceae, and Bacteroidaceae were less common in GDM pregnant women who followed the MNT for 2 weeks, while Bifidobacteriaceae and butyric acid-producing bacteria (Prevotellaceae and Lachnospiraceae) were more common than in GDM microbial samples at enrolment.

The GDM women recruited in Chen et al. [32••] had a proportion of carbohydrates of about 50–60%, and the remaining energy supplying nutrients contained 15–20% protein and 25–30% fat, with a reasonable distribution of cereals and potatoes, eggs, legumes, fish, dairy products, vegetables, and oils. The portions of cereals and potatoes, egg, beans, fish, and vegetables were evenly distributed over three meals, while milk and products were spread over 2–3 additional meals. Before the MNT, there were more *Rosella*, *Bifidobacterium*, *Clostridium*, *Holdemania*, and *Proteus* in the guts of pregnant women who adhered to the MNT. There were also more bacteria in the intestines of pregnant women who did not adhere to MNT recommendations. These bacteria, such as Leuconostocaceae, *Weissella*, *Prevotella*, and *Bacillus cereus*, help the body improve its blood sugar level. Pathogens like *Desulfovibrio*, *Aeromonas*, and *Gemella*, which can cause weight gain, poor glucose tolerance, and insulin resistance, were also more common in GDM pregnant women who did not adhere to their treatment plans.

The nutritional recommendation given to GDM women under Ferrocino et al. [23] study was 45% total energy from carbohydrates, < 10% of energy from rapidly absorbed sugars, 18–20% energy from protein, 35% energy from fats, at least 20–25 g/day fibre intake, and no alcohol intake. Ferrocino et al. reported a strong correlation between protein intake and *Faecalibacterium* in GDM pregnant women in the second and third trimesters [23]. *Alistipes*, on the other hand, revealed a positive correlation with fat intake after adjusting for weight and age of the GDM pregnant women [23]. They also observed a direct correlation between *Roseburia* and fibre consumption, although it did not meet the statistical thresholds.

## Discussion

This systematic review aimed to assess the effect of nutrition on gut microbiota modulation and its influence on metabolism in pregnant women. Several previous analyses examined the impact of lifestyle treatments on their ability to improve glucose control in pregnant women with GDM [33–35]. To the best of our knowledge, no systematic reviews are available on gut microbiota-targeted diets influencing the metabolic function of pregnant women. The evidence generated by this review demonstrates that dietary intake is associated with differences in the gut microbiota, which, in turn, is associated with metabolic health in pregnant women. If repeated in intervention study designs (i.e. randomized controlled trials and feeding studies), the current findings could contribute to more effective management targeting the risk of metabolic dysfunction in pregnant women.

**Fig. 2:**
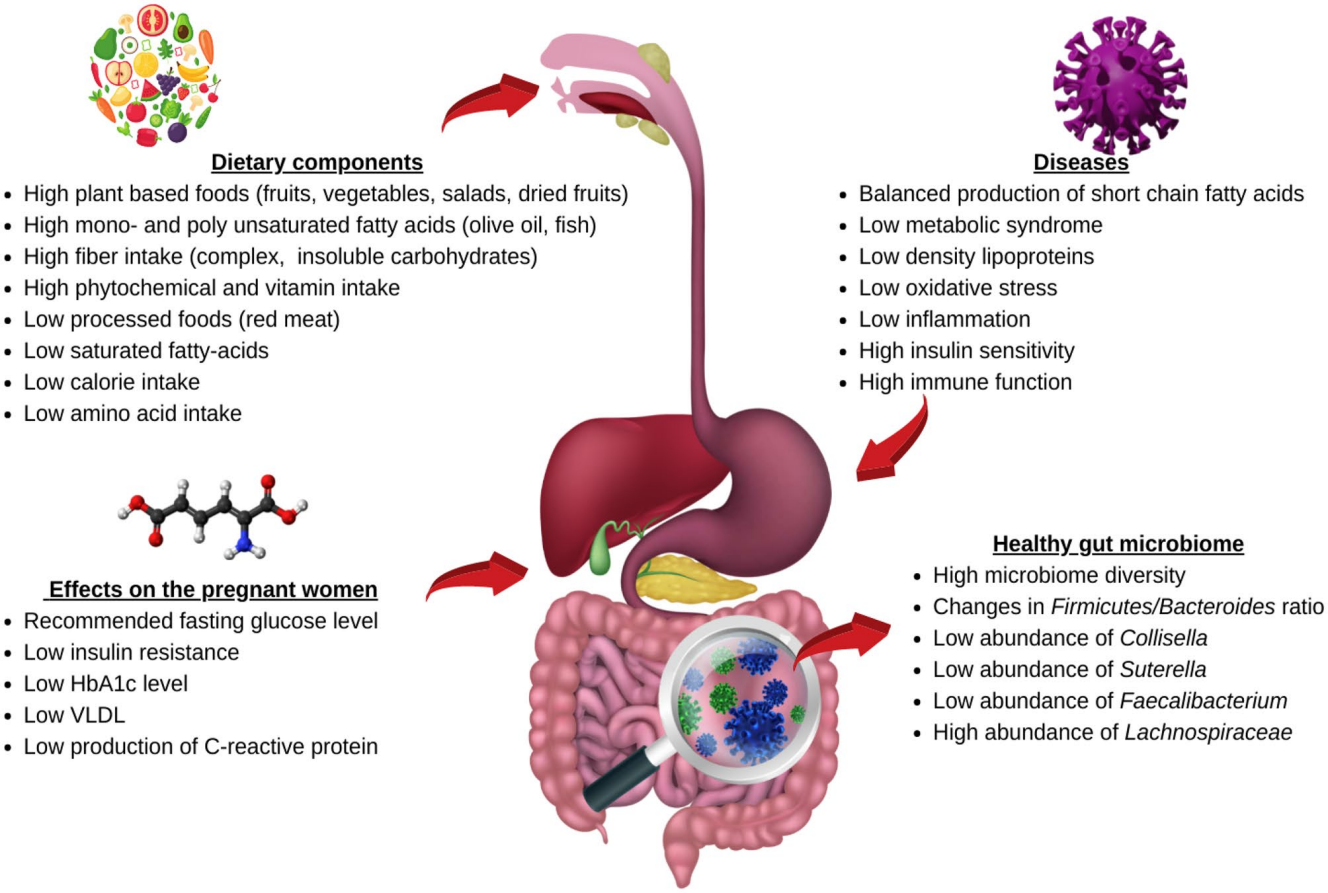
The role of diet in modifying gut microbes and its impact on the health and well-being of pregnant women. The figure was created and edited from the images available on Canva.com using Pro Content License

In this systematic review, a few gut microbes were related to the significance of a major nutrient intake and to metabolic markers in pregnant women (Fig. 2). Higher fibre intake was associated with a lower abundance of *Collisella* in healthy pregnant women, in the study by Barret et al. [25]. Similarly, researchers noticed a higher abundance of *Collisella* in oveweight or obese pregnant women [26] and pregnant women with GDM who did not adhere to MNT [23]. Dietary fibre is necessary for microbes to generate short-chain fatty acids (SCFAs) and to surpass the adipose tissue’s natural capacity for lipid storage rather than energy expenditure, resulting in a good energy balance that can significantly impact the gut microbe [36]. It decreases cholesterol absorption by increasing faecal mass and binding to bile acids in the digestive tract. [37–39]. Additionally, fermentation of dietary fibre has been demonstrated to decrease postprandial blood glucose and insulin levels [37]. Low fibre intake also promotes bacterial growth that can utilize host-secreted mucus glycoproteins and other “non-fibre” energy sources, which may influence the colonic mucus barrier and microbial overgrowth and proliferation [40].

*Collinsella*, on the other hand, is frequently referred to as a strictly anaerobic pathobiont that produces lactate (instead of butyrate or other SCFA). Increases in lactate amounts induce insulin resistance in skeletal muscle by suppressing glycolysis and impairing insulin signalling [41]. The present data suggest that a diet low in dietary fibre in pregnant women may influence the intestinal microbiota, particularly the *Collinsella* species. Gomez et al. [41] reported that changing *Collinsella* abundance may affect lipid and glucose metabolism and may serve as markers of poor glucose metabolism during pregnancy. As a result, we predict that decreased dietary fibre intake during early pregnancy results in abundant *Collinsella* spp., which disrupts gut integrity, resulting in increased inflammation and the beginning of maternal hyperglycaemia.

Secondly, high fibre and less carbohydrate consumption were related to the increased abundance of *Lachnospira* in healthy pregnant women and those with overweight/obesity and were negatively associated with very low-density lipoprotein (VLDL) cholesterol in these pregnant women [25, 28, 30••]. Barret et al. concluded that *Lachnospira* is favourably associated with vegetarian diets in their study since vegetarians consume less protein and sugar than omnivores, with modest changes in fatty acid intake [25] in healthy pregnant women. Similarly, Roytio et al. studied the connection between maternal gut microbiota and diet composition as determined by a 3-day food diary in overweight and obese pregnant women with varied fibre and fat intakes [28]. In their study, the high abundance of *Lachnospira* had an inverse relation with VLDL particles, triglyceride concentration, and total triglyceride levels [28]. In the same line, the Medika Study reported that several species of Lachnospiraceae were positively associated with fibre-, potassium-, and vegetable-based protein intakes in adults with chronic kidney disease [42, 43].

In contrast to these studies, high abundances of Lachnospiraceae were positively associated with glucose and/or lipid metabolism in women with obesity and metabolic syndrome [44], in individuals with glucose metabolism disorders [45], and in male patients [46], indicating the vital role of Lachnospiraceae in metabolic disturbance condition. *Roseburia*, one of the species under the Lachnospiraceae family, has also been elevated in obese pregnant women with ketonuria. However, the carbohydrate intake was not comparable to that of pregnant women without ketonuria [31].

According to a recently reviewed study, Lachnospiraceae species metabolize fermentable carbohydrates, i.e. dietary fibre, and create butyrate and other short-chain fatty acids that alter glucose metabolism and diabetes risk [47]. Metagenomics research has also demonstrated its ability to use complex organic materials and transport breakdown products of different sizes and components [48]. This was most likely accomplished through ATP binding cassette (ABC) transporter proteins expressed by the genomes of several Lachnospiraceae species [49]. However, the involvement of the beneficial Lachnospiraceae bacteria in high-fat/protein metabolism is less clear among pregnant women, leading to the controversial role of Lachnospiraceae between pregnant women and the adult population (older adults, individuals with metabolic diseases, and non-pregnant women). Further studies on this topic are required.

Thirdly, the pro-inflammatory-type bacteria, *Sutterella* (proteobacteria phylum), have been strongly correlated with high protein intake and C-reactive protein (CRP) levels in women with GDM [23]. Likewise, the predicted metagenomes revealed a link between *Sutterella* and KEGG genes involved in lipopolysaccharide production (LPS). Gram-negative bacteria (proteobacteria) can produce pro-inflammatory LPS, resulting in an inflammatory condition linked to type 2 diabetes and obesity [50]. Nevertheless, the linkage between high protein intake and increased abundance of *Sutterella* is undetermined in those pregnant women with GDM [23]. On the other hand, a high abundance of *Sutterella* was observed in overweight/obese pregnant women who had minimal consumption of iron supplements, according to the network analysis done by Nitert et al. [27]. The iron status could be essential in developing chronic inflammatory diseases [51]. Iron deficiency is associated with chronic inflammatory indicators and other well-known risk factors for diabetes, obesity, and metabolic syndrome [52–54]. Therefore, the evidence suggests that high protein intake and low iron levels lead to a high abundance of *Sutterella* in GDM and obese pregnant women. However, the conclusive mechanisms of this relationship remain unclear.

Diet therapy is widely assumed to influence the intestinal microbiota in GDM [55]. Metabolites generated from microbiota alter glucose homeostasis via intestinal gluconeogenesis [56]. Women who do not comply with a recommended dietary change (e.g. carbohydrate restriction) to treat GDM have a higher abundance of the Leuconostocaceae, *Weissella*, *Prevotella*, *Bacillus cereus*, and *Faecalibacterium*, implying that diet’s function on maternal blood sugar control may be mediated via the gut microbes [23, 32••]. Supporting that, Benno et al. reported that the intake of more meat, animal fat, sugar, processed foods, and low fibre foods (the usual westernized diet) is associated with a lower count of *Faecalibacterium prausnitzii*. In contrast, a high-fibre (vegetables and fruits) and low meat diet increase the count [57]. The increased abundance of *Faecalibacterium*, an anti-inflammatory bacterium, in a study done by Ferrocino et al., suggested that this rise may be a corrective mechanism to combat the pro-inflammatory condition that could harm the unborn [23]. Indeed, *Faecalibacterium* abundance and fasting glucose levels were found to have an inverse connection, considering the established relationship between inflammation and metabolic impairment. Accordingly, metagenomic investigations found *Faecalibacterium prausnitzii* highly discriminating in diagnosing type 2 diabetes [58, 59]. In human studies of faecal microbiota, these butyrate-producing bacteria were inversely related to diabetes [13, 60–62].

Wu et al. [24••] explored the ratio of Firmicutes to Bacteroidetes to comprehend the role of diet management during GDM pregnancy. A higher ratio of Firmicutes to Bacteroidetes was suggested as a possible biomarker of obesity and other metabolic syndromes compared with the normal BMI of pregnant women. The ratio of Firmicutes to Bacteroidetes changed differently in the GDM and non-GDM groups. At the end of the study, healthy samples with no diet management demonstrated an almost significant rise in the ratio of Firmicutes to Bacteroidetes, which suggests energy homeostasis changes during pregnancy. Zheng et al. [55] found that the Firmicutes/Bacteroidetes ratio was higher in the second (T2) trimester than in the first (T1) trimester, which was similar to what Wu et al. [24••] found in healthy pregnant women. In the study by Wu et al. [24••], the ratio of Firmicutes to Bacteroidetes did not change in GDM managed with diet intervention. This suggests that diet intervention could be helpful in GDM management  by affecting the Firmicutes/Bacteroidetes ratio.

Nevertheless, in a study by Ferrocino et al. [23], there was an increase in the number of Firmicutes and a decrease in the number of Bacteroidetes in MNT-adherent patients whose total and saturated fat intake and CRP levels went down during pregnancy. This could be because the patients gained excessive weight during pregnancy even though they were already overweight at the enrolment stage, which is nearly similar in obese people [63, 64••]. However, Chen et al. [32••] did not analyse and compare the ratio of Firmicutes/Bacteroides in obese women with GDM, which restricts us from reaching a conclusion. Thus, this theory has to be further explored to gain and update the knowledge on the influence of MNT on GDM intestinal microbiota.

There were substantial connections between different microorganisms’ macronutrients and metabolic function in all the studies analysed. Nevertheless, the gut microbiota field and its investigation in pregnant women are still in its early stages, and the precise mechanisms involved are difficult to be confirmed. Heterogeneity among individuals was a possible concern in the gut microbiota–diet analysis conducted. Research findings are hard to compare due to the diverse ethnicities and dietary habits of the researched cohorts, which result in multi-variations in the pattern of the intestinal microbiota, and the different methods used to analyse the microbiota, both of which produce sometimes contradicting findings. Moreover, to identify a significant effect of the treatment in this field, a sufficient sample size is required to account for the significant variability between studies reported on gut microbes.

## Conclusions

Lifestyle changes can alter gut flora and affect blood glucose homeostasis. To fully exploit the gut microbiota’s potency and influence on disease pathophysiology and health, research must examine a proper intervention timeframe, sample size, and long-term follow-up (throughout gestational duration) to thoroughly understand the relationship between the host and its microbes. We have previously demonstrated that altering the gut microbiota significantly affects the pathogenesis of GDM [39]. The current review supported the significant role of dietary intake in modifying gut microbiota composition and its influence on metabolic reactions among pregnant women. However, the implications of the current findings for every pregnant woman remained undetermined due to the diversity of food intake across cultures and ethnic groups worldwide. Another unsolved topic is why some people respond more profoundly to treatments than others. Research has focused mostly on preclinical models, restricting their translation into individuals for use as therapeutic approaches. The number of articles included in this systematic review limits a firm conclusion about the microbial species and metabolic outcomes. It emphasizes the importance of conducting further well-designed human trials. As the frequency of metabolic disorders and adverse obstetric outcomes grows, and our awareness of the gut microbes improves, there is an excellent opportunity to evaluate the relevance of certain bacteria and their activity within a given bacterial population. This, in return, will set the groundwork for transferring preclinical findings into clinical practice by combining numerous methodologies and individual features into a molecular genetics approach to give individually tailored lifestyle therapies.

## Supplementary Information

Below is the link to the electronic supplementary material.

Supplementary file1 (DOCX 26 KB)

Supplementary file2 (DOCX 18 KB)

Supplementary file3 (DOCX 22 KB)

Supplementary file4 (DOCX 18 KB)

Supplementary file5 (DOCX 15 KB)

## Data Availability

Data related to this systematic review are available within the article and as supplementary materials.
