# Supplementary material for: Diet Gut Microbiota Axis in Pregnancy: A Systematic Review of Recent Evidence
Source: Curr Nutr Rep. 2023 Feb 22;12(1):203–14. doi: 10.1007/s13668-023-00453-4 (PMC9974723; doi:10.1007/s13668-023-00453-4)
Supplement: Supplementary file 2 — Supplementary file2 (DOCX 18 KB) [file 13668_2023_453_MOESM2_ESM.docx]

**Diet-Gut Microbiota Axis in Pregnancy: A Systematic Review of Recent Evidence**

**Thubasni Kunasegaran, Vinod RMT Balasubramaniam, Valliammai Jayanthi T Arasoo, Uma Devi Palanisamy, Amutha Ramadas***

**Jeffrey Cheah School of Medicine and Health Sciences, Monash University Malaysia, 47500 Bandar Sunway, Malaysia**

***Amutha Ramadas (corresponding author)**

**Email: amutha.ramadas@monash.edu**

**Supplementary Table S2** Search strategy for all databases.

| **PubMed** | | |
| --- | --- | --- |
| **#** | **Query** | **Results** |
| 1 | (gestational diabetes mellitus) OR (gestational diabetes) OR (diabetes in pregnancy) | 22, 142 |
| 2 | (gut microbiota) OR (microbiome) OR (microbes) OR (microbial dysbiosis) OR (microbiota) | 129, 042 |
| 3 | (diet) OR (dietary intake) OR (nutrition) OR (fats) OR (high fat diet) OR (glycemic index) OR (carbohydrate) OR (fiber) OR (fibre) OR (vitamin) OR (vegetarian) OR (fruits) OR (vegetables) OR (protein) | 3,504,930 |
| 4 | (((gestational diabetes mellitus) OR (gestational diabetes) OR (diabetes in pregnancy) AND ((gut microbiota) OR (microbiome) OR (microbes) OR (microbial dysbiosis) OR (microbiota) AND ((diet) OR (dietary intake) OR (nutrition) OR (fats) OR (high fat diet) OR (glycemic index) OR (carbohydrate) OR (fiber) OR (fibre) OR (vitamin) OR (vegetarian) OR (fruits) OR (vegetables) OR (protein) | 202 |
| **Scopus** | | |
| **#** | **Query** | **Results** |
| 1 | (((gestational AND diabetes AND mellitus) OR (gestational AND diabetes) OR (diabetes AND in pregnancy) AND ((gut AND microbiota) OR (microbiome) OR (microbes) OR (microbial AND dysbiosis) OR (microbiota))) AND ((diet) OR (dietary AND intake) OR (nutrition) OR (fats) OR (high AND fat AND diet) OR (glycemic AND index) OR (carbohydrate) OR (fiber) OR (fibre) OR (vitamin) OR (vegetarian) OR (fruits) OR (vegetables) OR (protein))) | 245 |
| 2 | (((gestational AND diabetes AND mellitus) OR (gestational AND diabetes) OR (diabetes AND in pregnancy) AND ((gut AND microbiota) OR (microbiome) OR (microbes) OR (microbial AND dysbiosis) OR (microbiota))) AND ((diet) OR (dietary AND intake) OR (nutrition) OR (fats) OR (high AND fat AND diet) OR (glycemic AND index) OR (carbohydrate) OR (fiber) OR (fibre) OR (vitamin) OR (vegetarian) OR (fruits) OR (vegetables) OR (protein))) AND (EXCLUDE PUBYEAR, 2010) OR (EXCLUDE PUBYEAR, 2009) (EXCLUDE PUBYEAR, 2006) OR (EXCLUDE PUBYEAR, 2002) | 123 |
| **Ovid Medline** | | |
| **#** | **Query** | **Results** |
| 1 | (gestational diabetes mellitus or gestational diabetes or diabetes in pregnancy).mp.[mp=title, abstract, original file, name of substance word, subject heading word, floating sub-heading word, keyword heading word, organism supplementary concept word, protocol supplementary concept word, rare disease supplementary concept word, unique identifier, synonyms) | 17038 |
| 2 | (gut microbiota or microbiome or microbes or microbial dysbiosis or microbiota). mp.[mp=title, abstract, original file, name of substance word, subject heading word, floating sub-heading word, keyword heading word, organism supplementary concept word, protocol supplementary concept word, rare disease supplementary concept word, unique identifier, synonyms) | 114588 |
| 3 | (diet or dietary intake or nutrition or fats or high fat diet or glycemic index or carbohydrate or fiber or fibre or vitamin or vegetarian or fruits or vegetables or protein). mp.[mp=title, abstract, original file, name of substance word, subject heading word, floating sub-heading word, keyword heading word, organism supplementary concept word, protocol supplementary concept word, rare disease supplementary concept word, unique identifier, synonyms) | 5025810 |
| 4 | 1 and 2 and 3 | 64 |
| 5 | Limit 4 to yr= “2011-2022” | 54 |
| **Web of Science** | | |
| **#** | **Query** | **Results** |
| 1 | ALL=((((gestational diabetes mellitus) OR (gestational diabetes) OR (diabetes in pregnancy)) AND ((gut microbiota) OR (microbiome) OR (microbes) OR (microbial dysbiosis) OR (microbiota))) AND ((diet) OR (dietary intake) OR (nutrition) OR (fats) OR (high fat diet) OR (glycemic index) OR (carbohydrate) OR (fiber) OR (fibre) OR (vitamin) OR (vegetarian) OR (fruits) OR (vegetables) OR (protein))) | 285 |
| 2 | ALL=((((gestational diabetes mellitus) OR (gestational diabetes) OR (diabetes in pregnancy)) AND ((gut microbiota) OR (microbiome) OR (microbes) OR (microbial dysbiosis) OR (microbiota))) AND ((diet) OR (dietary intake) OR (nutrition) OR (fats) OR (high fat diet) OR (glycemic index) OR (carbohydrate) OR (fiber) OR (fibre) OR (vitamin) OR (vegetarian) OR (fruits) OR (vegetables) OR (protein))) and 2011 or 2012 or 2013 or 2014 or 2015 or 2016 or 2017 or 2018 or 2019 or 2020 or 2021 or 2022 (Publication Years) | 274 |
| 3 | ALL=((((gestational diabetes mellitus) OR (gestational diabetes) OR (diabetes in pregnancy)) AND ((gut microbiota) OR (microbiome) OR (microbes) OR (microbial dysbiosis) OR (microbiota))) AND ((diet) OR (dietary intake) OR (nutrition) OR (fats) OR (high fat diet) OR (glycemic index) OR (carbohydrate) OR (fiber) OR (fibre) OR (vitamin) OR (vegetarian) OR (fruits) OR (vegetables) OR (protein))) and 2011 or 2012 or 2013 or 2014 or 2015 or 2016 or 2017 or 2018 or 2019 or 2020 or 2021 or 2022 (Publication Years) and Review Articles or Meeting Abstracts or Proceedings Papers or Book Chapters or Editorial Materials (Exclude – Document Types) | 196 |
| **Cochrane library** | | |
| **#** | **Query** | **Results** |
| 1 | ((gestational diabetes mellitus) OR (gestational diabetes) OR (diabetes in pregnancy)):ti, ab, kw | 5724 |
| 2 | ((gut microbiota) OR (microbiome) OR (microbes) OR (microbial dysbiosis) OR (microbiota))):ti, ab, kw | 7420 |
| 3 | ((diet) OR (dietary intake) OR (nutrition) OR (fats) OR (high fat diet) OR (glycemic index) OR (carbohydrate) OR (fiber) OR (fibre) OR (vitamin) OR (vegetarian) OR (fruits) OR (vegetables) OR (protein)) :ti, ab, kw | 233711 |
| 4 | ((gestational diabetes mellitus) OR (gestational diabetes) OR (diabetes in pregnancy)):ti, ab, kw AND ((gut microbiota) OR (microbiome) OR (microbes) OR (microbial dysbiosis) OR (microbiota))):ti, ab, kw AND ((diet) OR (dietary intake) OR (nutrition) OR (fats) OR (high fat diet) OR (glycemic index) OR (carbohydrate) OR (fiber) OR (fibre) OR (vitamin) OR (vegetarian) OR (fruits) OR (vegetables) OR (protein)) :ti, ab, kw | 40 |
| 5 | ((gestational diabetes mellitus) OR (gestational diabetes) OR (diabetes in pregnancy)):ti, ab, kw AND ((gut microbiota) OR (microbiome) OR (microbes) OR (microbial dysbiosis) OR (microbiota))):ti, ab, kw AND ((diet) OR (dietary intake) OR (nutrition) OR (fats) OR (high fat diet) OR (glycemic index) OR (carbohydrate) OR (fiber) OR (fibre) OR (vitamin) OR (vegetarian) OR (fruits) OR (vegetables) OR (protein)) :ti, ab, kw  With Publication Year from 2011 to 2022, in Trials (word variations have been searched) | 39 |
