# Supplementary material for: Diet Gut Microbiota Axis in Pregnancy: A Systematic Review of Recent Evidence
Source: Curr Nutr Rep. 2023 Feb 22;12(1):203–14. doi: 10.1007/s13668-023-00453-4 (PMC9974723; doi:10.1007/s13668-023-00453-4)
Supplement: Supplementary file 3 — Supplementary file3 (DOCX 22 KB) [file 13668_2023_453_MOESM3_ESM.docx]

**Diet-Gut Microbiota Axis in Pregnancy: A Systematic Review of Recent Evidence**

**Thubasni Kunasegaran, Vinod RMT Balasubramaniam, Valliammai Jayanthi T Arasoo, Uma Devi Palanisamy, Amutha Ramadas***

**Jeffrey Cheah School of Medicine and Health Sciences, Monash University Malaysia, 47500 Bandar Sunway, Malaysia**

***Amutha Ramadas (corresponding author)**

**Email: amutha.ramadas@monash.edu**

**Supplementary Table S3** Studies that were excluded with reasons.

| **No** | **Citations** | **Reason for exclusion** |
| --- | --- | --- |
| 1 | Chen T, Zhang Y, Zhang Y, Shan C, Zhang Y, Fang K, et al. Relationships between gut microbiota, plasma glucose and gestational diabetes mellitus. J Diabetes Investig. 2021;12:641-50. https://doi:10.1111/jdi.13373. | Lack of dietary analysis |
| 2 | Crusell MKW, Hansen TH, Nielsen T, Allin KH, Ruhlemann MC, Damm, P, et al. Gestational diabetes is associated with change in the gut microbiota composition in third trimester of pregnancy and postpartum. Microbiome. 2018;15:89. https://doi:10.1186/s40168-018-0472-x. | Lack of dietary analysis |
| 3 | Cui M, Qi C, Yang L, Zhang M, Wang H, She G, et al. A pregnancy complication-dependent change in SIgA-targeted microbiota during third trimester. Food Funct. 2020;11:1513-24.https://doi:10.1039/c9fo02919b. | Lack of dietary analysis |
| 4 | Gao BB, Zhong MD, Shen Q, Wu Y, Cao MD, Ju SW, et al. Gut microbiota in early pregnancy among women with hyperglycaemia vs. normal blood glucose. BMC Pregnancy Childbirth. 2020;20:284. https://doi:10.1186/s12884-020-02961-5. | Lack of dietary analysis |
| 5 | Gomez-Arango LF, Barrett HL, McIntyre HD, Callaway LK, Morrison M, Dekker Nitert M. Connections between the gut microbiome and metabolic hormones in early pregnancy in overweight and obese women. Diabetes. 2016;65:2214-23. https://doi:10.2337/db16-0278. | Lack of dietary analysis |
| 6 | Hou M & Li F. Changes of intestinal flora, cellular immune function and inflammatory factors in Chinese advanced maternal age with gestational diabetes mellitus. Acta Medica Mediterranea. 2020;36:1137-42. | Lack of dietary analysis |
| 7 | Kuang YS, Lu JH, Li SH, Li JH, Yuan MY, He JR, et al. Connections between the human gut microbiome and gestational diabetes mellitus. Gigascience. 2017;6:1-12. https://doi:10.1093/gigascience/gix058. | Lack of dietary analysis |
| 8 | Li GX, Yin P, Chu SH, Gao WL, Cui SH, Guo SH, et al. Correlation analysis between gdm and gut microbial composition in late pregnancy. J Diabetes Res. 2021. 8892849.https://doi:10.1155/2021/8892849. | Lack of dietary analysis |
| 9 | Ma S, You Y, Huang L, Long S, Zhang J, Guo C, et al. Alterations in gut microbiota of gestational diabetes patients during the first trimester of pregnancy. Front Cell Infect Microbiol. 2020. https://doi:10.3389/fcimb.2020.00058. | Lack of dietary analysis |
| 10 | Mokkala K, Pellonpera O, Roytio H, Pussinen P, Ronnemaa T, Laitinen K. Increased intestinal permeability, measured by serum zonulin, is associated with metabolic risk markers in overweight pregnant women. Metabolism. 2016;69:43-50. https://doi:10.1016/j.metabol.2016.12.015. | Lack of dietary analysis |
| 11 | Mullins TP, Tomsett KI, Gallo LA, Callaway LK, McIntyre HD, Nitert MD, et al. Maternal gut microbiota displays minor changes in overweight and obese women with GDM. Nutr Metab Cardiovasc Dis. 2021;31:2131-39. https://doi:10.1016/j.numecd.2021.03.029 | Lack of dietary analysis |
| 12 | Abdullah B, Daud S, Aazmi MS, Idorus MY, Mahamooth MIJ. Gut microbiota in pregnant Malaysian women: a comparison between trimesters, body mass index and gestational diabetes status. BMC pregnancy and childbirth. 2022;21:152. https://doi:10.1186/s12884-022-04472-x. | Lack of dietary analysis |
| 13 | Sililas P, Huang L, Thonusin C, Luewan S, Chattipakorn N, Chattipakorn S, et al. Association between gut microbiota and development of gestational diabetes mellitus. Microorganisms. 2021;8:1686. https://doi:10.3390/microorganisms9081686. | Lack of dietary analysis |
| 14 | Wang J, Zheng J, Shi W, Du N, Xu X, Zhang Y, et al. Dysbiosis of maternal and neonatal microbiota associated with gestational diabetes mellitus. Gut. 2018;67:1614-25. https://doi:10.1136/gutjnl-2018-315988. | Lack of dietary analysis |
| 15 | Wang X, Liu H, Li Y, Huang S, Zhang L, Cao C, Baker PN, et al. Altered gut bacterial and metabolic signatures and their interaction in gestational diabetes mellitus. Gut Microbes. 2020;12:1-13. https://doi:10.1080/19490976.2020.1840765. | Lack of dietary analysis |
| 16 | Ye G, Zhang L, Wang M, Chen Y, Gu S, Wang K, et al. The gut microbiota in women suffering from gestational diabetes mellitus with the failure of glycemic control by lifestyle modification. J Diabetes Res. 2019;4598167. https://doi:10.1155/2019/6081248. | Lack of dietary analysis |
| 17 | Huang L, Sililas P, Thonusin C, Luewan S, Chattipakorn SC. Early gut dysbiosis could be an indicator of unsuccessful diet control in gestational diabetes mellitus. Journal of Diabetes. 2021; 13: 1054-58. https://doi:10.1111/1753-0407.13225. | Lack of dietary analysis |
| 18 | Su Y, Chen L, Zhang DY, Gan XP, Cao YN, et al. The characteristics of intestinal flora in overweight pregnant women and the correlation with gestational diabetes mellitus. Endocrine Connections 2021;10:1366-76. https://doi:10.1530/EC-21-0433. | Lack of dietary analysis |
| 19 | Su Y, Wang HK, Gan XP, Chen L, Cao YN, Cheng DC, et al. Alterations of gut microbiota in gestational diabetes patients during the second trimester of pregnancy in the Shanghai Han population. Journal of Translational Medicine. 2021;366. https://doi:10.1186/s12967-021-03040-9. | Lack of dietary analysis |
| 20 | Zheng W, Xu Q, Huang W, Yan Q, Chen Y, Zhang L, et al. Gestational diabetes mellitus is associated with reduced dynamics of gut microbiota during the first half of pregnancy. mSystems 2020;5:e00109-20. https://doi:10.1128/mSystems.00109-20. | Lack of dietary analysis |
| 21 | Zong-Jie L, Zhen C. Effects of metabolic syndrome on intestinal flora, inflammatory factors, and infants of pregnant patients. Clinical laboratory. 2020;66. https://doi:10.7754/Clin.Lab.2020.200226. | Lack of dietary analysis |
| 22 | Chu DM, Antony KM, Ma J, Prince AL, Showalter L, et al. The early infant gut microbiome varies in association with a maternal high-fat diet. Genome Med. 2016;8:77. https://doi:10.1186/s13073-016-0330-z. | Lack of maternal gut microbiota analysis |
| 23 | Lundgren SN, Madan JC, Emond JA, Morrison HG, Christensen BC, Karagas MR, et al. Maternal diet during pregnancy is related with the infant stool microbiome in a delivery mode-dependent manner. Microbiome. 2018;6:109. https://doi:10.1186/s40168-018-0490-8. | Lack of maternal gut microbiota analysis |
| 24 | Wu Y, Bible PW, Long S, Ming WK, Ding W, Long Y, et al. Metagenomic analysis reveals gestational diabetes mellitus-related microbial regulators of glucose tolerance. Acta Diabetol. 2020;57:569-81. https://doi:10.1007/s00592-019-01434-2. | Lack of maternal gut microbiota analysis |
| 25 | Laitinen K, Mokkala K. Overall dietary quality relates to gut microbiota diversity and abundance. Int J Mol Sci. 2019;20:1835. https://doi:10.3390/ijms20081835. | Lack of metabolic parameter analysis |
