# Supplementary material for: Diet Gut Microbiota Axis in Pregnancy: A Systematic Review of Recent Evidence
Source: Curr Nutr Rep. 2023 Feb 22;12(1):203–14. doi: 10.1007/s13668-023-00453-4 (PMC9974723; doi:10.1007/s13668-023-00453-4)
Supplement: Supplementary file 4 — Supplementary file4 (DOCX 18 KB) [file 13668_2023_453_MOESM4_ESM.docx]

**Diet-Gut Microbiota Axis in Pregnancy: A Systematic Review of Recent Evidence**

**Thubasni Kunasegaran, Vinod RMT Balasubramaniam, Valliammai Jayanthi T Arasoo, Uma Devi Palanisamy, Amutha Ramadas***

**Jeffrey Cheah School of Medicine and Health Sciences, Monash University Malaysia, 47500 Bandar Sunway, Malaysia**

***Amutha Ramadas (corresponding author)**

**Email: amutha.ramadas@monash.edu**

**Supplementary Table S4** Quality assessment of the prospective cohorts and cross-sectional studies (n=9)

|  | **Wu et al., 2022** | **Selma et al., 2021** | **Robinson et al., 2019** | **Ferrocino et al., 2018** | **Barret et al., 2018** | **Gomez-Arango et al., 2018** | **Mandal et al., 2016** | **Nitert et al., 2018** | **Roytio et al., 2017** |
| --- | --- | --- | --- | --- | --- | --- | --- | --- | --- |
| Q1 | Y | Y | Y | Y | Y | Y | Y | Y | Y |
| Q2 | Y | Y | Y | Y | Y | Y | Y | Y | Y |
| Q3 | NR | Y | NR | NR | NR | NR | NR | Y | Y |
| Q4 | Y | Y | Y | Y | Y | Y | NR | Y | Y |
| Q5 | N | N | N | N | N | N | N | Y | N |
| Q6 | Y | Y | N | Y | Y | N | Y | N | N |
| Q7 | N | Y | N | Y | N | N | Y | N | N |
| Q8 | Y | Y | Y | Y | N/A | Y | Y | Y | Y |
| Q9 | Y | Y | Y | Y | Y | Y | Y | Y | Y |
| Q10 | Y | N | N | Y | N | N | N | N | N |
| Q11 | Y | Y | Y | Y | Y | Y | Y | Y | Y |
| Q12 | NR | NR | NR | NR | NR | NR | NR | NR | NR |
| Q13 | N | N/A | N/A | N | N/A | N/A | Y | N/A | N/A |
| Q14 | N | Y | N | Y | N | Y | Y | N | Y |
| **Quality** | **Fair** | **Good** | **Fair** | **Good** | **Fair** | **Fair** | **Fair** | **Fair** | **Fair** |

The quality of included studies was assessed using the National Institutes of Health (NIH) Quality Assessment Tool for Observational Cohort and

Cross-Sectional Studies [14]. Q, question; CD, cannot be determined; NA, not applicable; NR, not reported; N, no; Y, yes.

**Q 1**. Was the research question or objective in this paper clearly stated? **Q 2.** Was the study population clearly specified and defined? **Q 3.** Was the participation rate of eligible persons at least 50%? **Q 4.** Were all the subjects selected or recruited from the same or similar populations (including the same time period)? Were inclusion and exclusion criteria for being in the study prespecified and applied uniformly to all participants? **Q 5.** Was a sample size justification, power description, or variance and effect estimates provided? **Q 6.** For the analyses in this paper, were the exposure(s) of interest measured prior to the outcome(s) being measured? **Q 7**. Was the timeframe sufficient so that one could reasonably expect to see an association between exposure and outcome if it existed? **Q 8**. For exposures that can vary in amount or level, did the study examine different levels of the exposure as related to the outcome (e.g., categories of exposure, or exposure measured as a continuous variable)? **Q 9.** Were the exposure measures (independent variables) clearly defined, valid, reliable, and implemented consistently across all study participants? **Q 10**. Was the exposure(s) assessed more than once over time? **Q 11**. Were the outcome measures (dependent variables) clearly defined, valid, reliable, and implemented consistently across all study participants? **Q 12.** Were the outcome assessors blinded to the exposure status of participants? **Q 13**. Was the loss to follow-up after baseline 20% or less? **Q 14.** Were key potential confounding variables measured and adjusted statistically for their impact on the relationship between exposure(s) and outcome(s).
