# Supplementary material for: Diet Gut Microbiota Axis in Pregnancy: A Systematic Review of Recent Evidence
Source: Curr Nutr Rep. 2023 Feb 22;12(1):203–14. doi: 10.1007/s13668-023-00453-4 (PMC9974723; doi:10.1007/s13668-023-00453-4)
Supplement: Supplementary file 5 — Supplementary file5 (DOCX 15 KB) [file 13668_2023_453_MOESM5_ESM.docx]

**Diet-Gut Microbiota Axis in Pregnancy: A Systematic Review of Recent Evidence**

**Thubasni Kunasegaran, Vinod RMT Balasubramaniam, Valliammai Jayanthi T Arasoo, Uma Devi Palanisamy, Amutha Ramadas***

**Jeffrey Cheah School of Medicine and Health Sciences, Monash University Malaysia, 47500 Bandar Sunway, Malaysia**

***Amutha Ramadas (corresponding author)**

**Email: amutha.ramadas@monash.edu**

**Supplementary Table S5** Quality assessment of case-control study (n=1)

|  | **Chen et al., 2022** |
| --- | --- |
| **Q1**. 1. Was the research question or objective in this paper clearly stated and appropriate? | Y |
| **Q2**. Was the study population clearly specified and defined? | Y |
| **Q3**. Did the authors include a sample size justification? | N |
| **Q4**. Were controls selected or recruited from the same or similar population that gave rise to the cases (including the same timeframe)? | Y |
| **Q5**. Were the definitions, inclusion and exclusion criteria, algorithms or processes used to identify or select cases and controls valid, reliable, and implemented consistently across all study participants? | Y |
| **Q6**. Were the cases clearly defined and differentiated from controls? | Y |
| **Q7**. If less than 100 percent of eligible cases and/or controls were selected for the study, were the cases and/or controls randomly selected from those eligible? | NR |
| **Q8**. Was there use of concurrent controls? | CD |
| **Q9.** Were the investigators able to confirm that the exposure/risk occurred prior to the development of the condition or event that defined a participant as a case? | CD |
| **Q10.** Were the measures of exposure/risk clearly defined, valid, reliable, and implemented consistently (including the same time period) across all study participants? | Y |
| **Q11.** Were the assessors of exposure/risk blinded to the case or control status of participants? | NR |
| **Q12.** Were key potential confounding variables measured and adjusted statistically in the analyses? If matching was used, did the investigators account for matching during study analysis? | N |
| **Quality** | **Good** |

Q, question; CD, cannot be determined; NA, not applicable; NR, not reported; N, no; Y, yes.
